# The glass ceiling perception and female teacher burnout: the mediating role of work–family conflict

**DOI:** 10.3389/fpsyg.2025.1551903

**Published:** 2025-04-14

**Authors:** Yun Wei, Geetha Subramaniam, Xueshen Wang

**Affiliations:** Faculty of Education, Language, Psychology & Music, SEGi University, Kota Damansara, Malaysia

**Keywords:** female teacher burnout, higher education institutions, JD-R theory, work–family conflict, glass ceiling, SDGs

## Abstract

**Introduction:**

This paper aims to investigate the mediating role of work–family conflict in the relationship between the perception of the glass ceiling and female teacher burnout. The glass ceiling can create significant career barriers for women, potentially increasing work–family conflict and, in turn, leading to higher levels of burnout. Exploring these dynamics can provide insights into the underlying mechanisms contributing to female teachers’ well-being and inform strategies to mitigate burnout in educational settings.

**Methods:**

The sample population comprises 200 female teachers working in higher educational institutions in China where data has been collected via a survey questionnaire, followed by SPSS and Smart PLS to analyse and test the hypotheses.

**Results:**

Results indicate that there is a positive relationship between the perception of glass ceiling, work–family conflict and female teacher burnout, and work–family conflict mediates the relationship between the perception of glass ceiling and female teacher burnout in higher educational institutions in China.

**Discussion:**

These findings highlight how work–family conflict caused by the glass ceiling perception negatively impacts burnout among female faculty members in higher educational institutions, which are relevant to the UN Sustainable Development Goals (SDGs) 3, 4, and 5 emphasizing women’s well-being, high-quality education, and gender equality, it offers actionable suggestions for policymakers to reduce the incidence of female teacher burnout.

## 1 Introduction

**Figure 1:**
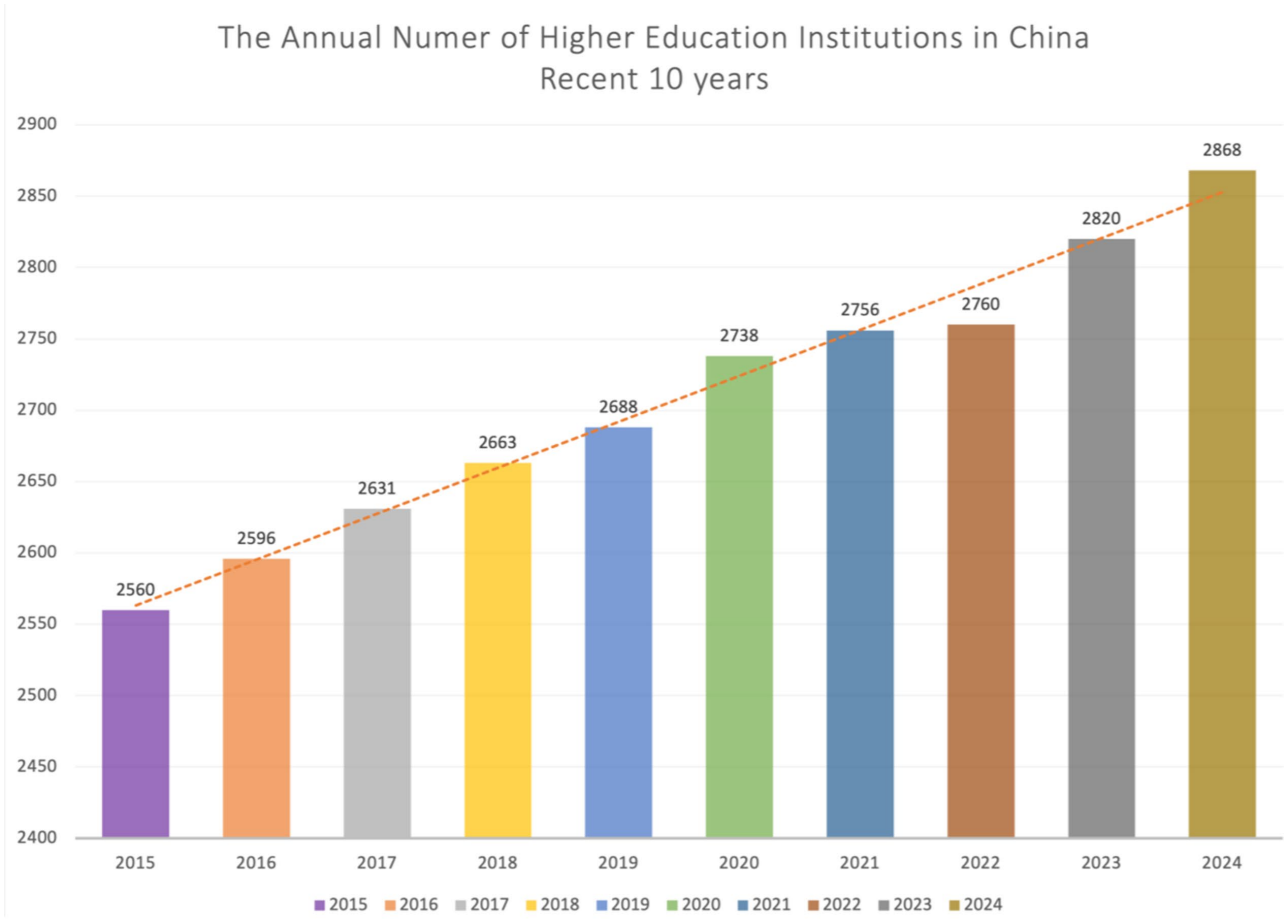
The annual number of higher education institutions in China (Ministry of Education, 2024a).

**Figure 2:**
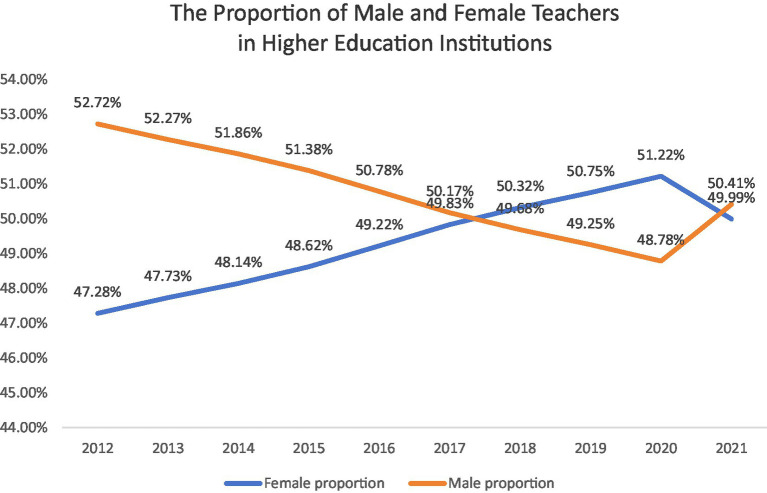
The proportion of female teachers in HEIs in China (Educational Statistics Yearbook of China, 2022).

In recent years, teacher burnout has been a major problem in academia, showing a rise in occurrences globally (Thomas and Reyes, 2024), especially following the COVID-19 pandemic, which intensified workload pressures for university faculty members (Zbanca et al., 2024). In addition, several studies have shown that female teachers in HEIs are more likely to suffer from burnout than male teachers. For example, an American university’s study revealed that female teachers (69%) reported higher levels of burnout than male teachers (57%) (Vyletel et al., 2023) and high levels of emotional weariness among teachers were also shown in a Spanish study, with female instructors experiencing burnout at a higher rate than male counterparts (Sánchez-Pujalte et al., 2021). Similarly, In China, teachers in higher education are facing severe job burnout (Cheng et al., 2023; Tan and Kim, 2024; Liu and Siththada, 2025). Over the past decade, the steady expansion of Chinese higher education institutions (HEIs) (Figure 1) has been accompanied by a significant increase in the proportion of female faculty members, with female teachers accounting for more than half of all faculty despite minor fluctuations (Figure 2). This shift highlights the growing presence of women in academia. However, despite this progress, female teachers continue to report higher levels of burnout compared to their male counterparts (Wu, 2021).

University instructors play a significant role in influencing young people’s academic performance and future employment (Valiente et al., 2011). However, According to Skaalvik and Skaalvik (2010), teaching has one of the highest rates of job burnout and is considered a high-risk profession due to the increasing social expectations and demands placed on teachers (Filho et al., 2021). Among these groups, university faculty members are particularly vulnerable, as they face unique pressures such as heavy workloads, research demands, and administrative responsibilities, which exacerbate their risk of burnout (Pan, 2023). Moreover, this stress can lead to many unfavorable outcomes such as decreased job commitment and satisfaction, impaired physical and mental health, and teacher turnover intention (Schaufeli and Enzmann, 2020). Even more concerning, there is evidence linking job exhaustion to a higher risk of suicidal thoughts and attempts (Freitas et al., 2023). Therefore, understanding the causes of burnout is crucial to reducing its detrimental effects.

In response to these adverse effects, the academic field is also trying to explore the causes of teacher burnout from multiple perspectives and reveal the deep mechanism of burnout. At present, the job demands-resources model (JD-R model), is often used as a conceptual framework to diagnose burnout’s possible causes of teacher burnout (Bakker et al., 2023). Based on JD-R model, the elements that contribute to teacher burnout can be divided into two broad categories: personal factors (i.e., optimism) and situational factors (i.e., workload). These two factors would differentiate between job demands such as unpleasant work environment and resources like resilience and social support (Bakker and Demerouti, 2017). It is worth noting that burnout is often triggered by work-related factors (job demands) rather than personal characteristics (Bakker et al., 2023). In other words, situational factors themselves can cause burnout, while certain personal factors can act as moderating variables that influence the extent to which work-related factors lead to burnout (Edú-Valsania et al., 2022).

**Figure 3:**
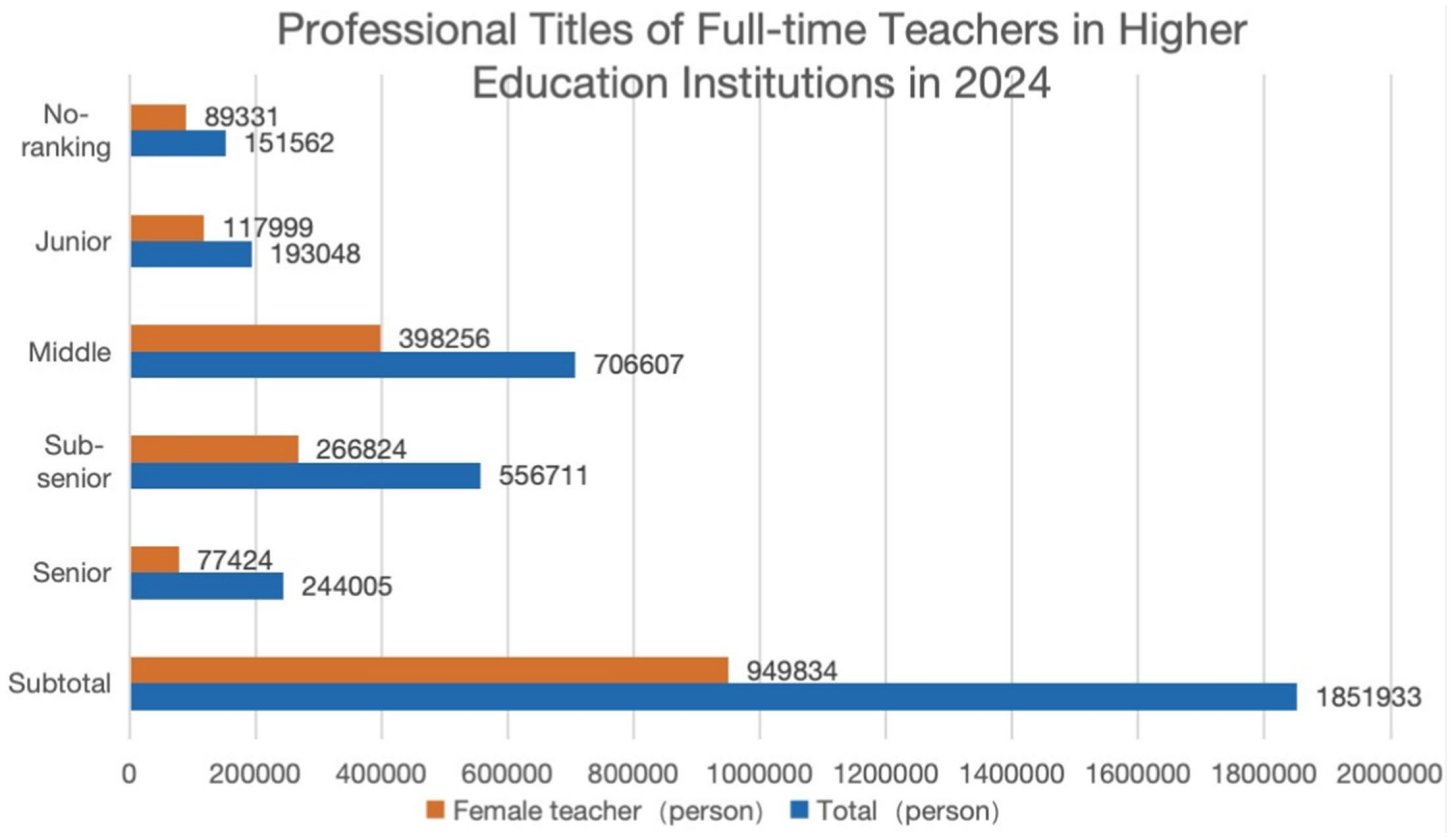
Professional titles of full-time teachers in HEIs in China (Ministry of Education, 2024b).

In the context of China, the glass ceiling, as a critical organizational job demand, is an often-overlooked factor contributing to female faculty burnout. As shown in Figures 2, 3, despite the increasing participation of female teachers in academia, they remain disproportionately concentrated in junior and middle professional titles, with limited access to senior ranks. This disparity suggests that numerical growth in female representation does not necessarily translate into equal opportunities in career advancement (Li et al., 2023), as many female teachers encounter structural barriers associated with the glass ceiling (Abbas et al., 2021). However, career stagnation caused by these barriers often leads to dissatisfaction, depression, stress and burnout (Taparia and Lenka, 2022). Similarly, work–family conflict, as another significant situational factor, exacerbates burnout among female faculty members. Balancing academic responsibilities with family obligations can create significant stress, particularly for women who often bear a disproportionate share of domestic duties (Greenhaus and Beutell, 1985; Su and Jiang, 2023). However, this work–family conflict is further exacerbated by the glass ceiling (Babic and Hansez, 2021), leading to increased frustration and emotional exhaustion (Pujol-Cols et al., 2025). Overall, the interaction between glass ceiling and work–family conflict underscores the complex challenges faced by female faculty in Chinese higher education. Addressing these barriers is crucial for promoting gender equity and reducing burnout in academia.

Therefore, this study focuses on two critical factors contributing to burnout among female faculty: work–family conflict and the glass ceiling. The relationship between these factors and burnout is highly complex and multifaceted, requiring in-depth exploration. The following literature review will examine their individual and combined effects on burnout in the context of Chinese higher education, shedding light on the intricate dynamics that underlie this phenomenon.

## 2 Literature review and hypotheses development

### 2.1 Glass ceiling perception

The phrase “glass ceiling” (GC) became widely recognized in 1986, describing invisible barriers that prevent individuals from advancing in organizations due to bias and discrimination (Srivastava et al., 2020; Ramos et al., 2022; Hossain, 2020). Gender Role Theory suggests that societal expectations and implicit biases further reinforce these barriers (Eagly and Karau, 2002), while Social Role Theory (Eagly et al., 1981) posits that workplace role are often assigned based on gender, disadvantaging women in career advancement. Sharma and Kaur (2019) explain that these invisible barriers hindering women’s career development exist across three dimensions: organizational, cultural, and social. Globally, the pursuit of Sustainable Development Goals (SDGs) has heightened attention to gender equality in academia, particularly in developing nations where systemic barriers impede women’s career progression. However, the glass ceiling phenomenon remains pervasive in HEIs (Khan et al., 2024). In China, it continues to limit female faculty members’ access to senior positions and leadership roles despite their increasing presence in academia (Bao and Yuan, 2024; Li et al., 2023). Many studies show that multiple factors contribute to the causes and effects of the glass ceiling, highlighting its complex nature (Shrestha et al., 2023; Ngaage et al., 2020).

For one thing, cognitive bias makes it difficult for women to advance in their careers, as they are often judged less favorably than men (Hussin et al., 2021). Men have long been seen as primary providers and more suited for leadership (Schneider and Bos, 2019), while women are expected to prioritize family responsibilities as mothers and housewives (Kongsomrarn et al., 2022). This traditional belief that women should stay home and rely on their husbands reinforce gender roles, creating bias and hindering their career advancement (Da Silva et al., 2022). Marital and childbearing status (third child policy) further affect hiring decisions (Imadoğlu et al., 2020; Fyall, and Gazley, B., 2015; Scalambrino and Lowery, 2017), forcing many women to delay or forgo career opportunities (Khalid and Aftab, 2023). However, this will make women self-doubt and lose confidence in their abilities in the long run (Williams, 2017). This loss of personal resources can result in detrimental psychological effects like stress, work–family conflict, and job burnout (D’sa et al., 2023; Bradshaw et al., 2024; Dubois-Shaik and Fusulier, 2017; Rr and Sathiyaseelan, 2019). For another, flawed promotion policies limit women’s opportunities for advancement (Eghlidi and Karimi, 2020; Sharma and Kaur, 2019). In Chinese universities, the “Promotion or out” culture adds stress through rigorous evaluation system based on research funding and publications (Lai and Li, 2020). The glass ceiling restricts access to critical resources like mentorship and career opportunities due to women’s dual burden at home and work or employers’ assumptions that marriage and motherhood reduce their availability (Pan, 2023). Overcoming these barriers requires extra time and effort to overcome these barriers (Hobfoll, 2011). To make matters worse, the collision between the three-child policy and the promotion-or-leave policy has put female teachers in a more difficult situation, exacerbating their overall strain (Luo, 2022; Li et al., 2023; Mittal and Kaur, 2023). Therefore, this persistent lack of access to promotions, unequal opportunities for research funding, and exclusion from decision-making bodies create a high-stress work environment, making the glass ceiling a significant job demand (Arora and Tiwary, 2024).

According to the JD-R model, the glass ceiling restricts job resources (self-confidence) while simultaneously increasing job demands (workload), thereby leading to emotional exhaustion and burnout (Herath et al., 2024; D’sa et al., 2023; French and Allen, 2020). However, shattering the glass ceiling can promote gender equality and give women equitable job opportunities (Sharma and Agarwal, 2024), which is consistent with SDG 5 aiming to empower women and girls at all levels and achieve gender equality (United Nations, 2020).

### 2.2 Work–family conflict

The term “work–family conflict” (WFC) is a type of role conflict that arises when a person’s commitment to one position depletes their ability to fulfill the obligations of other roles and it is divided into two directions (work–family conflict and family–work conflict) and three types: time, stress, and behavior based conflicts (Greenhaus and Beutell, 1985). When someone invests time and effort in one area, it becomes challenging for them to fulfill their obligations in another (Byron, 2005). In other words, a higher level of WFC, driven by stress, time constraints, or behavioral expectations, might result from a person’s level of involvement in the work domain (Huang et al., 2004). Conservation of Resources (COR) Theory (Hobfoll, 1989) suggests that stress occurs when people perceive a threat to their valuable resources or experience resource loss. Work–family conflict arises when competing demands drain time, energy, and emotional resources. Role Theory (Kahn et al., 1964) focuses on individual-level conflicts arising from juggling multiple roles and suggests that individuals occupy multiple roles (e.g., employee, parent, and spouse) that impose conflicting demands. When the expectations from work and family roles are incompatible, conflict arises.

Traditional gender norms and workplace pressure are significant factors that influence the conflict between work and family in China (Yang, 2016). It is worth noting that female teachers experience noticeably more work-family problems than their male counterparts in terms of behavior, energy, and time allocation (Gu, 2024; Ren and Caudle, 2020). Firstly, the long-standing Confucian tradition in China emphasizes women’s primary role as caregivers and homemakers, which continues to have an impact on working women in modern society (Fan et al., 2014). Also, three-child policy in China makes more women devote their time, feelings, and energy to raising their children and to assume greater responsibility in childcare due to gender roles. Raising three children shifts their focus toward family, reducing energy available for work (Luo, 2022). Thus, they devote more time to housework, childcare, and elder care than men do (Luo, 2025). Secondly, the demands of work, such as severe workloads and lengthy working hours, create pressure to meet research, teaching, and administrative duties while also managing household responsibilities. This results in a resource-depleting conflict (Pan, 2023; Pinto et al., 2024) since individuals cannot meet all of these expectations (Sieber, 1974). As a rule, female faculty members often face dual pressures from both professional and domestic roles, leading to higher resource consumption as they strive to meet societal and organizational expectations (Greenhaus and Beutell, 1985). Therefore, this phenomenon can lead to their burnout because their increased participation in the family can take away from their limited resources (time, strength, and attention to their profession), women are more negatively impacted by family interference at work, and as work–family conflicts intensify, burnout worsens (Allgood et al., 2022; Cerrato and Cifre, 2018).

From a JD-R perspective, WFC represents an additional job demand that further drains employees’ mental and emotional resources, amplifying burnout symptoms (Allgood et al., 2022). As a result, when they are faced with excessive demands from their jobs, university academics may experience significant work–family conflicts (Ogakwu et al., 2022). Additionally, when female educators face career advancement barriers, they often compensate by investing additional time and effort into their professional roles. This heightened work engagement, however, reduces the time and energy available for family responsibilities, intensifying WFC (Khan et al., 2024; Gu, 2024) or individuals often feel that missing out on promotion opportunities and being rejected can also lead to increased work–family conflict (Babic and Hansez, 2021).

### 2.3 Female teacher burnout

Burnout, initially defined by Freudenberger (1974), refers to physical, emotional, and mental exhaustion from prolonged exposure to emotionally demanding work. Maslach and Jackson (1981) later operationalized burnout as a multidimensional construct encompassing emotional exhaustion, depersonalization, and reduced personal accomplishment, which remains the dominant in burnout research. In the context of teaching, burnout is particularly relevant due to the high levels of interpersonal interaction and emotional labor required (Maslach et al., 2001). According to Hanju and Shiquan (2024), teacher burnout is defined as the feelings and behaviors exhibited by teachers when they are under significant strain at work and engaged in frequent interpersonal conflicts over time. However, there is no unified definition of teacher burnout in the literature, as its manifestations may vary across educational contexts and populations. Accordingly, this study adopts the Job Demands-Resources (JD-R) (Bakker and Demerouti, 2007), conceptualizing teachers’ burnout as a state of chronic physical and emotional depletion caused by prolonged work stress (Tan and Kim, 2024).

In this study, the JD-R model explains how the interplay between job demands and job resources influences burnout. Excessive job demands, such as work–family conflict, deplete individuals’ resources, while a lack of job resources, such as career advancement opportunities exacerbates this process (Demerouti et al., 2001; Demerouti and Bakker, 2023). However, the glass ceiling perception functions as a key factor within this framework, simultaneously increasing job demands and restricting access to critical job resources. On the one hand, individuals facing the glass ceiling often experience stress from organizational culture and leadership, which creates barriers to career progression (Soumya and Sathiyaseelan, 2021). On the other hand, work–family conflict arises from both internal pressure, such as personal ambitions for career advancement, and external demands imposed by institutions (Lin et al., 2015). In other words, the pressure caused by the glass ceiling perception happens to be one of the important factors of work–family conflict. This stress will exacerbate the contradiction between work and family, thereby causing work–family conflict (O’Neill and Follmer, 2020).

Specifically, career stagnation caused by the glass ceiling perception depletes personal resources, compelling female faculty to invest additional time and effort to break through these barriers (Dashper, 2020). They experience heightened job demands in the form of longer working hours, extra responsibilities, and the need to demonstrate higher performance to compensate for perceived professional disadvantages, which increases psychological stress and decreases the amount of time and effort available for family obligations (Gu, 2024; Babic and Hansez, 2021; Huang et al., 2004). Simultaneously, the glass ceiling perception limits access to supportive job resources, such as mentorship, promotions, and institutional support (Scalambrino and Lowery, 2017), making it even more challenging to cope with these demands. This imbalance between high job demands and insufficient resources intensifies work–family conflict and depletes personal energy, ultimately leading to burnout (Uslukaya et al., 2025). Additionally, the glass ceiling perception lowers an employee’s degree of job satisfaction and organizational commitment and this type of strain can sometimes even be transferred to the family, leading to family strife and work–family conflicts (Tiwari et al., 2019; Wu et al., 2003). Thus, within the JD-R model, the glass ceiling perception acts as a structural constraint that amplifies burnout by both raising job demands and restricting vital career resources.

In addition to JD-R model, conservation of resources theory (COR) (Hobfoll, 1989) also supports the view that glass ceiling perception increases the WFC. According to this theory, women educators who face the glass ceiling (i.e., insufficient availability of information, guidance, social support, coaching from supervisors, learning opportunities, performance evaluations, and promotions) will use more resources to succeed, but this process of trying to make up for the loss of resources by using them is actually a form of stress that can drain a person’s resource reserve, making them devote more time and energy to their work, which leaves them with less time for their families, leading to physical and psychological health. These detrimental effects have an adverse effect on how people perform at home, exacerbating work–family conflict and resulting in burnout (Hobfoll, 1989).

Previous studies on teacher burnout have primarily focused on individual, social, and organizational factors, with less attention to gender-specific barriers such as the glass ceiling or the mediating role of work–family conflict (WFC) in this relationship (Babic and Hansez, 2021). Moreover, most studies have treated teachers as a homogeneous group, overlooking gender differences. In China, research on teacher burnout have largely concentrated on primary and secondary school educators (Tan and Kim, 2024), while university faculty, despite their heavy workloads and significant professional pressures, have often been overlooked. However, female faculty in HEIs face distinct challenges in academia, and their experiences of burnout deserve independent examination. To address this gap, this study employs the job demands-resources (JD-R) model to examine how the glass ceiling influences burnout among female faculty in higher education institutions (HEIs) and whether work–family conflict mediates this effect. To enhance comprehension of the correlation between them, the following theory and hypotheses are proposed.

Finally, the line of reasoning above has led to the formulation of the following hypothesis.

*H1*: The perception of GC has a positive relationship with FTB.

*H2*: WFC has a positive relationship with FTB.

*H3*: WFC mediates the relationship between the perception of GC and FTB.

## 3 Methods

### 3.1 Participants

In this study, the sample consisted of two hundred participants who are dispersed throughout different parts of Jiangxi province, China because sample sizes should be between more than 30 and fewer than 500 in order to be considered suitable for research studies (Mandeville and Roscoe, 1971). Jiangxi Province was chosen as the research site due to three factors. Firstly, Jiangxi has a large and growing number of higher education institutions, with the number of universities increasing each year. This expansion has led to heightened work demands and academic pressures on faculty members, making it a relevant setting to explore burnout. Secondly, previous studies on teacher burnout in China have largely focused on economically advanced regions, while research on burnout in less developed areas like Jiangxi remains limited. Studying Jiangxi helps fill this gap and offers a more balanced understanding of burnout among female university faculty in different regional contexts. Lastly, practical considerations such as access to participants and institutional support ensured efficient and reliable data collection, further justifying Jiangxi as an appropriate research site.

Additionally, questionnaires, along with a cover letter guaranteeing confidentiality, were directly distributed to the respondents. This study was only open to female teachers in HEIs and participation was completely voluntary. A response rate of 93.8% was achieved from the 221 completed questionnaire returned out of the 253 distributed. After removing surveys with incomplete or missing data, 200 surveys (response rate: 89.8%) were kept for further analysis.

### 3.2 Measures

The following scales have been adapted according to research demands and are all 5-point Likert-type scale, using with anchors 1 (strongly disagree) to 5 (strongly agree).

#### 3.2.1 Glass ceiling perceptions

We measured the perception of GC using nine items (Appendix 1 from the Women Workplace Culture Questionnaire (WWC) (Bergman and Hallberg, 2002). This scale can be utilized in studies and assessments of the working conditions for women in relation to micro inequalities and psychosocial barriers that are significant for women’s health (Bergman, 2003). Example items include: “Women have to be more accomplished in their work than men in order to be promoted”; and “Women receive more unfair judgements of their work performance than men” (α = 0.87).

#### 3.2.2 WFC

Work and Family Conflict Scale (WAFCS) was developed by Haslam et al. (2015) (Appendix 2). There are 10 items (two dimensions): five WFC items (Family badly impacted by work) and five FWC items (Work is adversely affected by family involvement). Example items include: “My work prevents me spending sufficient quality time with my family”; and “It is difficult to concentrate at work because I am so exhausted by family responsibilities”(α = 0.90).

#### 3.2.3 FTB

Burnout was assessed with eight items (Appendix 3) from the Maslach Burnout Inventory—Educators Survey (MBI-ES) (Maslach et al., 1996). This scale was developed especially for teachers, administrators, staff, volunteers, and other educators who operate in educational environments and it has been widely utilized in the literature (Cao et al., 2024). Example items include: “I feel emotionally drained from my work”; and “I worry that this job is hardening me emotionally”(α = 0.89).

### 3.3 Data analysis

After giving serious thought to the design of the study and the characteristics of the sample, SPSS (Statistical Package for the Social Sciences) programme was used for descriptive analysis while SmartPLS 4.0 software was used to evaluate the hypotheses (Inferential Analysis) through Partial Least Squares-Structural Equation Modeling (PLS-SEM). According to Hair et al. (2021), PLS-SEM is more appropriate for social science research when the goal is theory expansion. Mediation analysis was conducted after evaluating the measurement and structural models during the data analysis. To measure perceptions and attitudes, a reflective measurement approach is the most common and suitable; therefore, all constructs were measured as first-order reflective constructs.

## 4 Results

### 4.1 Demographic profile

As can been seen from Table 1, 31.5% of female teachers were aged between 41 and 50 years and the majority of them (47%) were married, with 41% having no children. 37% of the respondents had a Master’s Degree and 40.5% were lecturers. The percentage of female teachers with 11–20 years of experience and those with more than 20 years was the same (31.5%). Most (31%) of the respondents had a pre-tax monthly income between RMB 8,001 and RMB 11,000. Most (54%) of the respondents came from public universities. Table 1 highlights a diverse range of demographic information among the respondents.

**Table 1:** Respondents’ profile.

Demographic factors	Frequency (*n*)	Percentage (%)
Age		
Below 30	44	22.0
31–40	37	18.5
41–50	63	31.5
51 and above	56	28.0
Marital Status		
Single	67	33.5
Married	94	47.0
Divorced	27	13.5
Widowed	12	6.0
No. of Children		
0	82	41.0
1	56	28.0
2	35	17.5
More than 2	27	13.5
Highest Educational Level		
Bachelor’s Degree	65	32.5
Master’s Degree	74	37.0
Doctoral degree	61	30.5
Academic Ranking		
Associate Lecturer	34	17.0
Lecturer	81	40.5
Associate Professor	60	30.0
Professor	25	12.5
Years of Teaching		
1–3 years	30	15.0
4–10 years	44	22.0
11–20 years	63	31.5
Above 20 years	63	31.5
Monthly Income (Pre-tax)		
Under RMB 5000	34	17.0
RMB 5000–RMB 8000	50	25.0
RMB 8001–RMB 11000	62	31.0
RMB 11001 and above	54	27.0
Type of Institution		
Public University	108	54.0
Private University	92	46.0
Total	200	100

**Figure 4:**
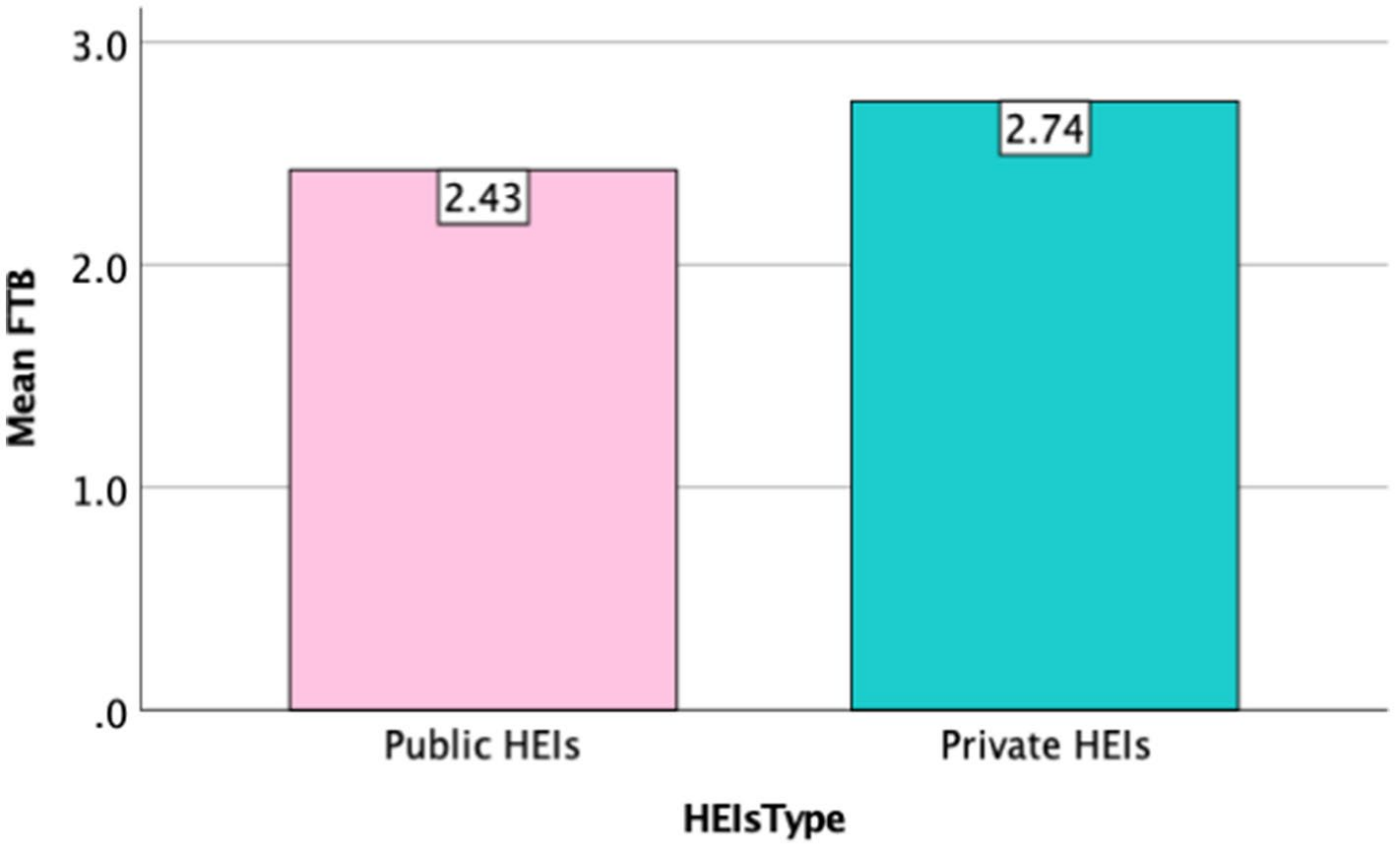
Mean of HEIs type.

However, it is worth noting that institution types have different effects on female teacher burnout. Figure 4 illustrates the mean levels of female teacher burnout (FTB) across different types of higher education institutions (HEIs). The x-axis represents HEI types, categorized into Public and Private HEIs, while the y-axis indicates the mean FTB values. Female teachers in private HEIs experience a higher burnout level of 2.74, compared to 2.43 for those in public HEIs. This suggests that burnout in China is more prevalent among teachers in private institutions than in public ones.

These findings align with Akhtar and Saleem (2020), who found that teachers in public educational institutions generally enjoy greater job security compared to those in private settings. The precarious nature of employment in private HEIs can be a constant source of stress, contributing to higher burnout rates among faculty members in private institutions. The threat of job insecurity may lead to feelings of inadequacy and anxiety, exacerbating the onset of burnout symptoms. This is also supported by Ptáček et al. (2019), stating that individuals who received the greatest financial rewards reported the lowest levels of burnout. This suggests that financial pressures can significantly impact wellbeing, particularly in private institutions where workload demands and salary expectations may not be met. Additionally, Wang (2023) pointed out that in China, teachers at private institutions frequently experience high levels of stress due to their increased workloads, job demands, and lack of job security. These factors raise the likelihood of burnout among professors in private institutions.

Jiangxi’s private higher education institutions are characterized by high job instability, heavy workloads, and limited resources, as they often rely on tuition fees rather than government funding. Faculty members, especially female teachers, face intense pressure to meet performance standards while receiving lower salaries and fewer benefits compared to public university staff. In contrast, female teachers in public universities experience greater job security, stable salaries, and better welfare benefits, reducing financial and professional stress. Additionally, stronger institutional support and clearer career advancement paths provide public university teachers with a sense of stability and professional fulfillment. These factors make female teachers in public institutions less prone to burnout compared to their counterparts in Jiangxi’s private universities.

### 4.2 Common method variance

To address potential biases, we implemented both statistical and procedural remedies. First, Harmon’s single-factor test was conducted on three study variables since all respondents in this study came from the teaching profession in HEIs in China. The results showed that the latent factor explained 41.5% of the variance (Table 2). Since the threshold for this test is 50%, the results indicate that common method bias was found to be negligible (Kock, 2015). In addition, considering all construct responses were gathered at the same time, making the survey susceptible to common method bias, additional statistical tests were performed to further validate the results. The variance inflation factor (VIF) was examined, with values ranging from 1.766 to 2.366 (Table 3), all below the benchmark of 3.3, indicating that there is no significant common method bias (Hair et al., 2019). Finally, procedural remedies such as ensuring respondent anonymity and emphasizing that there were no right or wrong answers were applied to further minimize potential response bias.

**Table 2:** Harmon’s single-factor test.

	Initial eigenvalues			Extraction sums of squared loadings		
Component	Total	% of Variance	Cumulative%	Total	% of Variance	Cumulative%
1	11.192	41.450	41.450	11.192	41.450	41.450
2	3.104	11.495	52.945	3.104	11.495	52.945
3	1.993	7.382	60.327	1.993	7.382	60.327

**Table 3:** Measurement model result.

Construct/items	Loadings	α	CR	AVE	VIF
Perception of glass ceiling		0.915	0.916	0.596	
PGC_1	0.772				2.040
PGC_2	0.781				2.184
PGC_3	0.749				1.993
PGC_4	0.814				2.366
PGC_5	0.762				1.945
PGC_6	0.799				2.198
PGC_7	0.785				2.145
PGC_8	0.728				1.772
PGC_9	0.755				1.863
Work–Family Conflict		0.922	0.924	0.587	
WFC_1	0.743				1.918
WFC_2	0.719				2.125
WFC_3	0.774				2.246
WFC_4	0.797				2.017
WFC_5	0.765				2.033
WFC_6	0.774				2.365
WFC_7	0.808				2.075
WFC_8	0.772				2.138
WFC_9	0.770				1.902
WFC_10	0.735				1.766
Female Teacher Burnout		0.909	0.911	0.612	
FTB_1	0.787				2.061
FTB_2	0.736				1.836
FTB_3	0.777				2.005
FTB_4	0.793				2.167
FTB_5	0.779				1.996
FTB_6	0.801				2.174
FTB_7	0.810				2.267
FTB_8	0.772				1.995

### 4.3 Measurement model

The measurement model of this study was assessed by measuring Indicator Reliability (outer loading), Internal Consistency Reliability (Cronbach’s alpha values), Convergent Validity (Composite Reliability and Average Variance Extracted) and Discriminant Validity values (HTMT). All relevant values in this study were higher than the minimum benchmarks, which are shown in the Table 4. Among this, outer loading values ranged from 0.719 to 0.814 while there was a range of 0.909 to 0.922 for Cronbach’s alpha and 0.911 to 0.924 for composite reliability (CR). The AVE values were between 0.587 and 0.612 and HTMT values were all less than 0.9. The details are in Tables 3 and 5.

**Table 4:** Criteria value.

Category	Threshold value	Sources
Outer Loading	≥0.7	Chin (1998)
Cronbach’s Alpha (α)	0.95 ≥ 0.7	Cronbach (1951)
Composite Reliability (CR)	0.95 ≥ 0.7	Hair et al. (2019)
Average Variance Extracted (AVE)	≥0.5	Hair et al. (2019)
Heterotrait-Monotrait (HTMT)	<0.9	Henseler et al. (2015)

**Table 5:** Discriminant validity HTMT ratios.

Construct	FTB	PGC	WFC
Female Teacher Burnout			
Perception of glass ceiling	0.642		
Work–Family Conflict	0.633	0.498	

Based on the above results, we can conclude that the measurement items were reliable and internally consistent, with no confusion or overlap between constructs. The model performs very well, demonstrating both good convergent and discriminant validity, making it suitable for subsequent structural equation analysis.

### 4.4 Structural model

**Figure 5:**
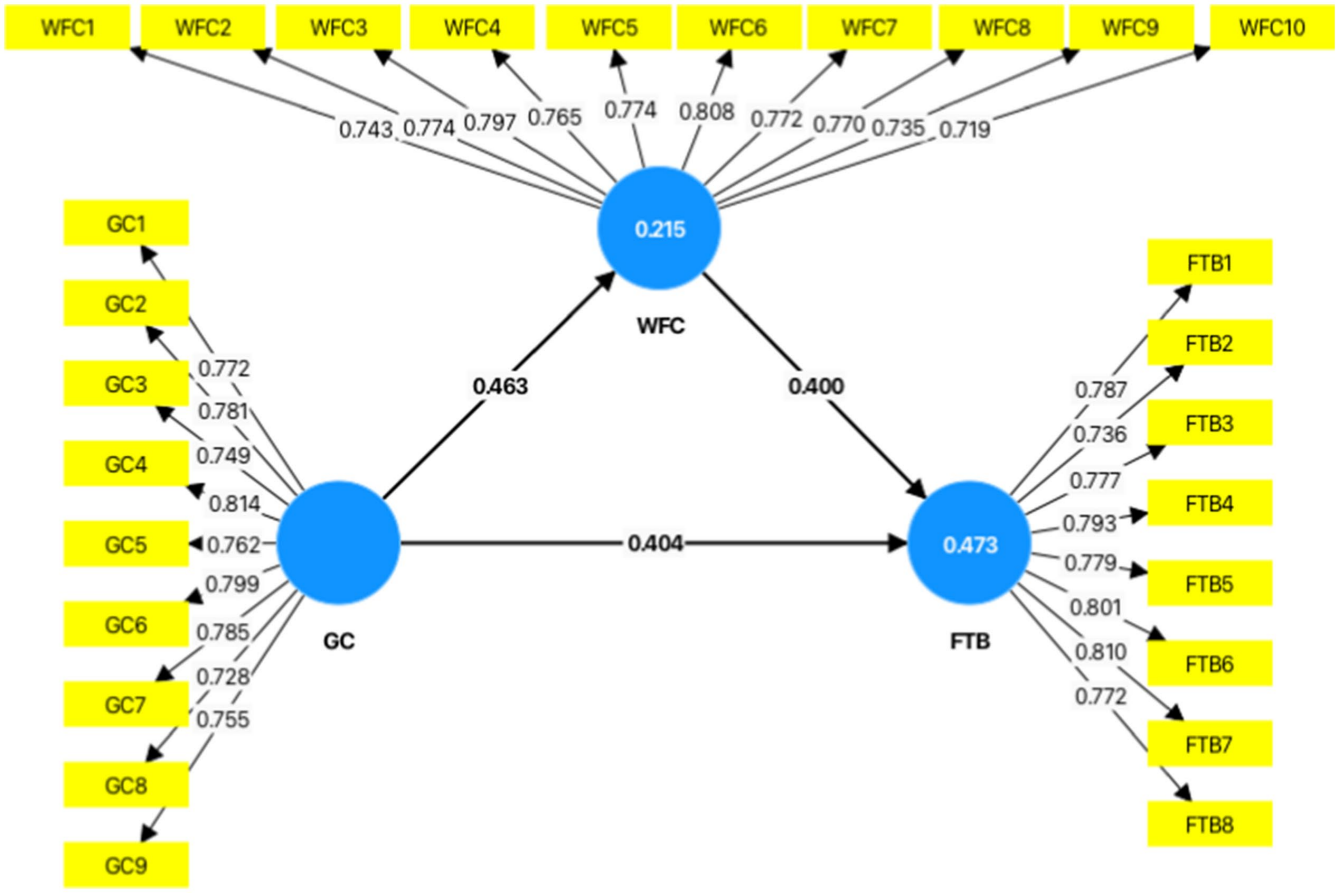
Path model.

Now that the constructs’ validity and reliability have been demonstrated, we look at the model’s structural element, also known as the inner model. In this part, significance and relevance of the path coefficients, the level of R^2^ and the predictive relevance Q^2^ values were used to assess the structural model. A crucial measure for evaluating the suitability of a structural model is the coefficient of determination (R2), which represents the percentage of explained variation in the endogenous latent variables and model’s in-sample predictive power (Hair et al., 2011; Rigdon, 2012). Weak, modest, moderate, and great explanatory power are shown by R2 values ranging from 0 to 0.10, 0.11 to 0.30, 0.30 to 0.50, and > 0.50, respectively (Hair and Alamer, 2022). Besides, Q2 assesses the predictive power of a structural model. For an endogenous latent construct to be considered sufficiently predictively relevant, its Q^2^ value must be sufficiently different from zero (Stone, 1974; Geisser, 1974). Q^2^ values greater than 0, 0.25, and 0.50 mean small, medium and large prediction (Hair et al., 2019). In this study, the results of R2, Q2 and path coefficients are in the following Tables 6 and 7, followed by a path model diagram (Figure 5).

**Table 6:** Structural model assessment.

Constructs	R^2^	Q^2^	Results
WFC	0.215	0.120	Modest/small
FTB	0.473	0.283	Moderate/medium

**Table 7:** Results of the path analysis.

Path	Path coefficient	*t*-value	*p*-value	95% Confidence Interval [2.5, 97.5%]	Results
GC -> FTB	0.404	6.923	0.000	[0.291, 0.518]	*H1*:Supported
WFC -> FTB	0.400	7.352	0.000	[0.293, 0.506]	*H2*:Supported
GC -> WFC -> FTB	0.185	5.885	0.000	[0.130, 0.252]	*H3*:Supported

As can be seen from Table 6, on the one hand, the range of the R^2^ values was 0.215 to 0.473. Given the study’s setting and the quantity of predictor constructs, the R^2^ value of WFC was weak but acceptable (Hair et al., 2019) while the R^2^ value of FTB was moderate. On the other hand, the Q^2^ value of WFC and FTB are all more than zero, meaning meaningful and results show small and medium for each (Hair et al., 2019).

The significance of hypotheses is displayed in Table 7. This study’s research model introduced three hypotheses. Results from the bootstrapping analysis showed that all of the hypotheses had strong support and each of them will be covered in the section that follows:

*H1:* The perception of GC has a positive relationship with FTB.

Table 7 shows that the first hypothesis posits a positive relationship between the perception of GC and FTB, and was supported by the data (β = 0.404, *p* < 0.01). suggesting that the relationship is statistically significant and provides strong evidence against the null hypothesis (i.e., there is a significant effect when T > 1.96, *p* < 0.05). The 95% confidence interval does not include zero, further confirming the significance of the relationship (Hair et al., 2014). More specifically, higher perceptions of GC are significantly linked with higher FTB.

This notion is supported by D’sa et al. (2023), who argue that the glass ceiling can exacerbate feelings of emotional exhaustion and overwhelm, which can result in burnout. Moreover, these findings are in accordance with the past research that the existence of the glass ceiling impairs female employees’ job performance by resulting in burnout, stress and discontent (Khan and Ferdoos, 2024; Taparia and Lenka, 2022; Mert and Levent, 2020; Lekchiri et al., 2019).

*H2:* WFC has a positive relationship with FTB.

Table 7 shows that the second hypothesis posits a positive relationship between WFC and FTB, and was supported by the data (β = 0.400, p < 0.01), demonstrating that the effect of WFC on FTB is statistically significant and substantial support is found for the hypothesis. The 95% confidence interval does not include zero, further confirming the significance of the relationship. More specifically, increases in WFC are associated with higher levels of burnout among female teachers.

The findings conform to the study conducted by Liu et al. (2024) and Koşarsoy and Özgül (2024), showing that WFC is positively associated with burnout including three dimensions. This result also aligns with the studies indicating that work–family conflict in the teaching context might result in pressures that would cause exhaustion and reducing work–family conflicts is essential for reducing occupational burnout among teachers (Trillo et al., 2024; Li et al., 2024).

*H3:* WFC mediates the relationship between the perception of GC and FTB.

Table 8 shows that the third hypothesis posits WFC as a mediating variable in the relationship between the perception of GC and FTB, and this was supported by the data from indirect effects, total effects and mediating effect.

**Table 8:** Specific indirect effect with WFC as a mediator.

Path	Path coefficient	Mean	Standard deviation	*t*	*p*	95% Confidence interval
GC -> WFC -> FTB	0.185	0.188	0.031	5.885	0.000	[0.130, 0.252]

#### 4.4.1 Indirect effects with WFC as a mediator

The indirect effect of WFC as a mediating variable is shown in Table 8. GC (β = 0.185, *p* = 0.000) has a significant positive indirect effect on FTB through WFC. The 95% confidence interval does not include zero (0.130, 0.252), further confirming the significance of the relationship. It was established that the perception of GC exerts an indirect influence on FTB through the mediation of WFC. More specifically, higher perceptions of GC are perceived to increase work–family conflict, which in turn leads to higher levels of burnout.

#### 4.4.2 Total Effect with WFC as a Mediator

When WFC is considered a mediator, the total effect of GC on FTB is shown in Table 9. GC (β = 0.589, p = 0.000) has a significant total effect on FTB. The 95% confidence interval does not include zero, further confirming the significance of the relationship (0.497, 0.680). This indicates that higher perceptions of the glass ceiling are strongly associated with increased female teacher burnout, both directly and indirectly through work–family conflict.

**Table 9:** Total effects of WFC as a mediator.

Path	Path Coefficient	Mean	Standard Deviation	*t*	*p*	95%Confidence Interval
GC -> FTB	0.589	0.594	0.047	12.538	0.000	[0.497, 0.680]

In summary, when WFC serves as a mediator, the indirect effect and total effect of GC on FTB are all significant, suggesting that the association between the perception of GC and FTB was proven to be mediated by WFC.

#### 4.4.3 Strength of mediating effect of WFC

Table 10 presents the mediating effect of WFC as a mediator. The mediation analysis revealed that WFC partially mediated the relationship between GC and FTB. The variance accounted for (VAF) was 31.4%, indicating a partial mediation effect. This suggests that while WFC plays a significant role in explaining the relationship, GC still has a direct influence on FTB.

**Table 10:** Mediating effect of WFC.

Hypotheses	IV	MV	DV	Direct effect	Indirect effect	Total effect	VAF	Results
H3	GC	WFC	FTB	0.404 (6.923)	0.185 (5.885)	0.589 (12.538)	31.4%	Partial Mediation

The findings of this study support the that the glass ceiling perception negatively influences teacher burnout through mediating role of work–family conflict in HEIs in China. These findings are aligned with Khan and Khan (2022) who found that glass ceiling perception negatively affects people who struggle with work-family problems. Subordinates were given fewer promotions at the time because supervisors thought their performance was below average (Javadizadeh et al., 2022; Mistry et al., 2024). Additionally, evidence for this is found in various studies that have demonstrated how work–family conflict serves as a mediating variable in the relationship between the perception of GC and other factors such as turnover intention, career success, well-being, job engagement and job satisfaction (Mistry et al., 2024; Khan and Khan, 2022; Babic and Hansez, 2021; Saleem and Mateou, 2024). Also, the findings of this study are consistent with previous studies showing that work–family conflict plays a mediating role between other factors such as workload, stress, techno stress and burnout (Wang et al., 2024; Yuan et al., 2023; Rahman and Singh, 2024).

## 5 Discussion

This study aims to determine the impact of GC and WFC on FTB in HEIs in China. In a competitive economy, women now make up half of the human capital (Zidan and Fathy, 2020) and the sustainable development of female teachers plays a pivotal role in promoting the development of world-class universities (Li et al., 2023; Iqbal et al., 2022). However, although they make considerably more contributions than males, they are nonetheless unable to achieve and rise to high-level positions due to invisible hurdles like glass ceilings. In particular, female faculty in higher education are traditionally underrepresented and face many challenges in the workplace that can negatively affect their health and well-being. Thus, this study highlights the detrimental effects of these barriers by developing a theoretical framework based on JD-R theory. After examining empirical data and testing three hypotheses, the results show that the perception of GC is positively associated with burnout and this relationship is mediated by WFC. Related research on this finding is discussed below.

The first finding of this study is that glass ceiling perception causes teacher burnout, which is consistent with studies by D’sa et al. (2023), Khan and Ferdoos (2024), Taparia and Lenka (2022), Mert and Levent (2020). Lieberman et al. (2018) and Öztürk and Şimşek (2019) revealed that female teachers in education are less likely than men to hold lower positions in colleges, more of them work in temporary jobs, and they make less money than men with similar skills. In spite of their accomplishments and abilities in job growth, the imperceptible and unbreakable barrier that keeps women from rising to positions of higher academic standing, decision-making authority, or upper management. In addition to JD-R model, the conservation of resources theory (COR) states that people have a finite amount of personal resources (including time, energy, and emotions) that need the greatest amount of work to get, protect, and maintain (Hobfoll et al., 2018). However, the structural barriers imposed by the glass ceiling force women to invest more resources than their male counterparts to achieve similar career progress, often without proportional rewards or recognition due to structural factors, cultural and social norms, implicit bias in the professional environment (Hussin et al., 2021; Khalid and Aftab, 2023; Kongsomrarn et al., 2022; Da Silva et al., 2022). Accordingly, employers’ physical and mental resources are depleted by glass ceiling perception can lead to burnout (D’sa et al., 2023; Bradshaw et al., 2024). In addition, to break the glass ceiling in universities, institutions should promote awareness of gender biases, ensure fair workload distribution, enforce anti-discrimination policies, implement objective performance evaluations, and support women’s professional development. Strengthening legal protections and fostering a culture of equality can help advance female faculty’s career growth (Luo, 2022).

The second finding is that work–family conflict (WFC) has a positive effect on teacher burnout, which is supported by Liu et al. (2024), Koşarsoy and Özgül (2024), Trillo et al. (2024) and Li et al. (2024). Ren and Caudle (2020) found that Chinese academic women bear more family responsibilities, particularly in parenting, leading to gendered academic career paths. Even highly educated academic women are not exempt from the deeply embedded national norms in China regarding women’s roles in childcare and family. Despite China’s dual-income family structure, males are still typically seen as breadwinners, while women shoulder more domestic duties. Accordingly, this imbalance drains women’s resources both at home and at work, making it harder to manage both roles effectively. Similarly, traditional gender norms in Western societies intensify WFC for women more than men (Cerrato and Cifre, 2018). Men often view housework as optional rather than obligatory, further increasing women’s burden (Khan et al., 2024). WFC not only strains women’s psychological and emotional resources but also limits their ability to meet work demands, accelerating burnout (Iqbal et al., 2025; Liu et al., 2024). In addition, French et al. (2018) and khan and khan (2022) emphasize the strong link between support and work–family conflict. Positive organizational support lowers the degree of work–family conflict, enabling people to get past imperceptible obstacles and take advantage of chances for professional growth (Kurtessis et al., 2017). When workers feel supported, they gain confidence and seize opportunities. Constructive feedback and open discussions help them reassess their job boundaries and maintain self-esteem (Cheng and Yi, 2018). The negative effects of job demands on health and well-being can be mitigated or mitigated by job resources (i.e. self-esteem, confidence) (Demerouti and Bakker, 2023; Shin and Hur, 2021). However, GC and WFC, which lead to job stress (Soumya and Sathiyaseelan, 2021; Etemadinezhad et al., 2024), are considered job demands that affect burnout.

The third finding reveals that glass ceiling (GC) perceptions exacerbate teacher burnout through work–family conflict (WFC) via a sequential psychological process. Perceive career stagnation (GC) initially triggers profound frustration and injustice (Khan and Khan, 2022), leading to compensatory over commitment either through intensified work efforts (e.g., volunteering for extra administrative duties) or heightened family obligations (e.g., increased childcare following China’s three-child policy; Luo, 2022). These compensatory behaviors fundamentally reshape role dynamics, creating severe WFC through time-based conflict as competing demands exceed available hours, strain-based conflict through emotional spillover between domains, and behavior-based conflict from incompatible role expectations (Greenhaus and Beutell, 1985). The sustained WFC then systematically depletes psychological resources (Hobfoll, 1989), first eroding emotional regulation capacity through chronic stress (Babic and Hansez, 2021), then diminishing self-efficacy as repeated balancing attempts fail, and finally prompting social withdrawal from support networks (Maslach and Leiter, 2016).

This resource depletion process is particularly acute in China’s high-pressure academic environment where the three-child policy’s gendered burdens intersect with long work hours and limited recovery opportunities, creating perfect storm conditions for burnout. The mechanism aligns with the effort-recovery model (Meijman et al., 1998), wherein GC-induced WFC prevents psychological detachment from work, making burnout virtually inevitable when combined with perceived career barriers. This explanatory pathway is empirically robust, with cross-cultural studies consistently identifying WFC as the critical mediator between GC perceptions and burnout (Mistry et al., 2024; Wang et al., 2024; Yuan et al., 2023; Saleem and Mateou, 2024; Rahman and Singh, 2024).

On the contrary, some studies present different views. The finding that the GC contributes to WFC contrasts with studies by Chen et al. (2021) and Khan and Ferdoos (2024) which suggests that WFC is a predictor of the glass ceiling. WFC consumes personal time and energy, leaving fewer resources for career growth, which in turn reinforces GC barriers and ultimately leads to burnout. Lin et al. (2018) stated that WFC prevents them from overcoming imperceptible boundaries and makes them indifferent to their professional growth and success. Hence, WFC lowers their promotion chances (Hoobler et al., 2010). The discrepancy between this study’s findings and existing literature may be due to the contextual and cultural differences. This study finds that the GC itself acts as a primary barrier that intensifies WFC, ultimately resulting in burnout. In the Chinese higher education system, institutional barriers such as limited promotion opportunities, gender biases, and lack of support force female faculty to work longer hours and expend more effort to prove their competence. This additional strain reduces their time and energy for family responsibilities, heightening WFC and exacerbating burnout. Thus, rather than WFC being the initial cause, this study suggests that GC creates the conditions that make work–family conflict more severe, leading to burnout.

In fact, the glass ceiling is often studied in career advancement but rarely linked to burnout, especially in higher education. While most research highlights its impact on professional growth, its psychological toll, leading to stress and burnout, is overlooked. In academia, GC perception not only restricts career opportunities but also worsens work–family conflict, creating a cycle of frustration and emotional exhaustion. In China, traditional cultural norms and gender role expectations make this issue more pressing. Therefore, this intersection between the glass ceiling, work–family conflict, and burnout presents a new area for research. Additionally, understanding how GC and WFC contribute to burnout is essential for developing interventions aimed at fostering a healthier and more equitable academic landscape for female educators. Addressing female teacher burnout can enhance the well-being and education quality (Pressley et al., 2024; Taylor et al., 2024),while promoting gender equity (Huck, 2022). Though burnout research has advanced, challenges for female faculty persist. The rise in female educators does not mean historical gender biases have disappeared or that their struggles in academia have been resolved (Demerouti et al., 2021).

### 5.1 Theoretical implication

The results of this study contribute to the existing literature on burnout among female teachers in several key ways. First, it extends the Job Demands-Resources (JD-R) theory by exploring the relationship between the perception of GC and burnout, introducing gendered organizational factors as a critical job demand influencing burnout. Second, it fills a gap in the literature by examining how glass ceiling perceptions specifically affect female teachers, an area that has been underexplored in the context of burnout. Finally, the study clarifies the mediating role of (WFC), showing how WFC exacerbates FTB, particularly when influenced by gendered barriers such as the glass ceiling. These contributions deepen our understanding of the mechanisms driving burnout in female faculty and highlight the importance of addressing both gender inequality and work–family conflict in reducing burnout.

### 5.2 Practical implication

The findings of this study offer valuable implications for multiple stakeholders. HEIs play a crucial role in promoting gender equality by fostering inclusive academic environments, supporting female faculty, and addressing structural inequalities (Chankseliani and McCowan, 2021). Given that burnout often results from a combination of high job demands and insufficient job resources (Demerouti et al., 2021), HEIs should reassess workplace policies and optimize job features. To effectively address this issue, targeted interventions should be customized to meet the needs of female teachers.

Firstly, highly burned-out teachers should prioritize fewer demanding tasks that align with their strengths and seek social support from colleagues, family or professional networks, as this can help reduce stress and work–family conflict. Secondly, administrators should ensure fair workload distribution, implement flexible schedules, and enforce reasonable work hours to prevent unpaid overtime and minimize work encroaching on personal time. Thirdly, institutions play a key role in creating clear promotion pathways, reducing non-teaching duties, and supporting mentorship, leadership training, and family-friendly policies (i.e., childcare support, mental health services) to support female faculty’s career progression. Lastly, policy makers should implement gender-equity policies, such as transparent promotion criteria, leadership quotas, and flexible work arrangements, to help break the glass ceiling and foster a more inclusive academic environment.

By implementing these targeted strategies, mitigating female teacher burnout aligns with the United Nations’ Sustainable Development Goals (SDGs). Educational institutions can promote gender equality (SDG 5), ensure good health and well-being (SDG 3), and contribute to quality education (SDG 4). A balanced and supportive system not only reduces burnout and enhances fairness but also fosters sustainable career development for female teachers, ultimately driving social stability and economic growth in alignment with the Sustainable Development Goals (SDGs).

### 5.3 Limitations and future research

This study has three limitations that should be acknowledged. Firstly, the focus on female teachers in Chinese higher education institutions limits the generalizability of the findings to other regions or populations. Secondly, the study examines limited variables, excluding other potential confounding variables (e.g., institutional policies or support systems) that may influence the relationships between constructs. Thirdly, the reliance on cross-sectional data restricts the ability to establish causal relationships or capture the dynamic nature of burnout over time.

To address these limitations, future research could consider the following directions. First, adopting longitudinal designs could provide deeper insights into how burnout evolves over time and identify causal relationships between work–family conflict, the glass ceiling, and burnout. Second, incorporating qualitative approaches such as interviews or focus groups, could offer a more nuanced understanding of female teachers’ lived experiences. Similarly, conducting a mixed-method approach that combines both quantitative and qualitative data can also offer a more comprehensive perspective. Third, conducting cross-cultural comparisons would help assess the generalizability of the findings and identify context-specific interventions that effectively address teacher burnout in diverse educational systems. Finally, future studies could add some confounding variables, such as institutional policies or support systems, to better explain the complex dynamics of burnout.

## 6 Conclusion

This study adopts a localized perspective on burnout, focusing on female teachers in HEIs in China and the mechanisms through which work–family conflict and the glass ceiling contribute to burnout. In Chinese higher education, academic women already hold a significant position, and this position is only expected to grow in the years to come. By focusing specifically on female university instructors, this study extends burnout research beyond the general teacher population and highlights the impact of gender-specific career barriers on occupational well-being. Additionally, the findings demonstrate the mutual reinforcement between these factors, emphasizing the urgent need to address career barriers and work–family conflict to mitigate burnout risks. By identifying these relationships, the study contributes theoretically by extending the JD-R model to the context of female academics, offering a nuanced understanding of burnout drivers. Practically, it underscores the importance of institutional policies and these measures can enhance faculty well-being and retention while fostering a more equitable and productive academic environment.

## Data Availability

The original contributions presented in the study are included in the article/Supplementary material, further inquiries can be directed to the corresponding author.
