# Corrigendum: The glass ceiling perception and female teacher burnout: the mediating role of work–family conflict

**DOI:** 10.3389/fpsyg.2025.1612846

**Published:** 2025-05-13

**Authors:** Yun Wei, Geetha Subramaniam, Xueshen Wang

**Affiliations:** Faculty of Education, Language, Psychology & Music, SEGi University, Kota Damansara, Malaysia

**Keywords:** female teacher burnout, higher education institutions, JD-R theory, work–family conflict, glass ceiling, SDGs

In the published article, the Funding statement was incorrect. The statement originally stated, “The author(s) declare that no financial support was received for the research and/or publication of this article.” The corrected Funding statement appears below.

