# Supplementary material for: The glass ceiling perception and female teacher burnout: the mediating role of work–family conflict
Source: Front Psychol. 2025 Apr 14;16:1551903. doi: 10.3389/fpsyg.2025.1551903 (PMC12035654; doi:10.3389/fpsyg.2025.1551903)
Supplement: Supplementary file 1 [file Supplementary_file_1.docx]

**Appendix 1: Work and Family Conflict Scale (WAFCS)**

Source: Haslam et al. (2015)

Instructions: Please answer the following questions about yourself by indicating the extent of your agreement using the following Likert scale.

| Strongly Disagree | Disagree | Neutral | Agree | Strongly Agree |
| --- | --- | --- | --- | --- |
| 1 | 2 | 3 | 4 | 5 |

| **No.** | **Dimension** | **WORK-FAMILY CONFLICT** | **Likert Scale** | | | | |
| --- | --- | --- | --- | --- | --- | --- | --- |
| 1 | WFC | My work prevents me spending sufficient quality time with my family. | 1 | 2 | 3 | 4 | 5 |
| 2 |  | My family misses out because of my work commitments. | 1 | 2 | 3 | 4 | 5 |
| 3 |  | My work has a negative impact on my family life. | 1 | 2 | 3 | 4 | 5 |
| 4 |  | Working often makes me irritable or short tempered at home. | 1 | 2 | 3 | 4 | 5 |
| 5 |  | There is no time left at the end of the day to do the things I’d like at home (e.g., chores and leisure activities). | 1 | 2 | 3 | 4 | 5 |
| 6 | FWC | My family will prevent me from being a better employee. | 1 | 2 | 3 | 4 | 5 |
| 7 |  | My work performance suffers because of my personal and family commitments. | 1 | 2 | 3 | 4 | 5 |
| 8 |  | It is difficult to concentrate at work because I am so exhausted by family responsibilities. | 1 | 2 | 3 | 4 | 5 |
| 9 |  | Family related concerns or responsibilities often distract me at work. | 1 | 2 | 3 | 4 | 5 |
| 10 |  | My family has a negative impact on my day to day work duties. | 1 | 2 | 3 | 4 | 5 |

**Appendix 2: The Women Workplace Culture Questionnaire (WWC)**

Source: Bergman and Hallberg (2002)

Instructions: Please answer the following questions about yourself by indicating the extent of your agreement using the following Likert scale.

| Strongly Disagree | Disagree | Neutral | Agree | Strongly Agree |
| --- | --- | --- | --- | --- |
| 1 | 2 | 3 | 4 | 5 |

| **No.** | **Glass Celling** | **Likert Scale** | | | | |
| --- | --- | --- | --- | --- | --- | --- |
| 1 | Women have fewer opportunities than men for professional development at the workplace. | 1 | 2 | 3 | 4 | 5 |
| 2 | Women receive more unfair judgements of their work performance than men. | 1 | 2 | 3 | 4 | 5 |
| 3 | Working life is characterized by a negative attitude towards women. | 1 | 2 | 3 | 4 | 5 |
| 4 | Superiors have a negative attitude towards women. | 1 | 2 | 3 | 4 | 5 |
| 5 | It is more difficult for women than men to "be themselves" at work. | 1 | 2 | 3 | 4 | 5 |
| 6 | Women have less employment security than men. | 1 | 2 | 3 | 4 | 5 |
| 7 | Women's speeches at meetings are often ignored by men. | 1 | 2 | 3 | 4 | 5 |
| 8 | Women have to be more accomplished in their work than men in order to be promoted. | 1 | 2 | 3 | 4 | 5 |
| 9 | Women are less assertive compared to men to obtain fair compensation promotion or opportunities for professional development. | 1 | 2 | 3 | 4 | 5 |

**Appendix 3: Maslach Burnout Inventory—Educators Survey (MBI-ES)**

Source: Maslach et al. (1996)

Instructions: Please answer the following questions about yourself by indicating the extent of your agreement using the following Likert scale.

| Strongly Disagree | Disagree | Neutral | Agree | Strongly Agree |
| --- | --- | --- | --- | --- |
| 1 | 2 | 3 | 4 | 5 |

| **No.** | **TEACHER BURNOUT** | **Likert Scale** | | | | |
| --- | --- | --- | --- | --- | --- | --- |
| 1 | I feel emotionally drained from my work. | 1 | 2 | 3 | 4 | 5 |
| 2 | Working with people all day long requires a great deal of effort. | 1 | 2 | 3 | 4 | 5 |
| 3 | It stresses me too much to work in direct contact with people. | 1 | 2 | 3 | 4 | 5 |
| 4 | I feel used up at the end of the workday. | 1 | 2 | 3 | 4 | 5 |
| 5 | I feel I treat students as if they were impersonal“ objects. ” | 1 | 2 | 3 | 4 | 5 |
| 6 | I worry that this job is hardening me emotionally. | 1 | 2 | 3 | 4 | 5 |
| 7 | I really don't care about what happens to some of my students. | 1 | 2 | 3 | 4 | 5 |
| 8 | I have become more insensitive to people since I've been working. | 1 | 2 | 3 | 4 | 5 |

**References**

Bergman, B., and Hallberg, L. R. M. (2002). Women in a male-dominated industry: factor analysis of a women workplace culture questionnaire based on a grounded theory model. *Sex Roles* 46, 311–322. doi: 10.1023/A:1020276529726

Haslam, D., Filus, A., Morawska, A., Sanders, M. R., and Fletcher, R. (2015). The work–family conflict scale (WAFCS): development and initial validation of a self-report measure of work–family conflict for use with parents. *Child Psychiatry Hum. Dev.* 46, 346–357. doi: 10.1007/s10578-014-0476-0

Maslach, C., Jackson, S. E., and Leiter, M. P. (1996). *Maslach burnout inventory manual*. Palo Alto,California, USA: Consulting Psychologists Press.
